# Diagnostic Performance and Agreement of MST and NUTRISCORE Compared with GLIM Criteria in Ambulatory Cancer Patients: Results from the OncoNutridos Study

**DOI:** 10.3390/nu18091452

**Published:** 2026-04-30

**Authors:** Carmen Ripa, Olatz Olariaga, Sara Vallinas, Mariola Sirvent, Larraitz Leunda, Elena Prado, Rosa Romero-Jimenez, Laia Pérez-Cordón, Paloma Terroba, Sara Hernández, Amelia Chica, Rocio Gázquez, Fernando Quintana, Isabel Caba, Maria Encina García, on behalf of the OncoNutridos Group Study

**Affiliations:** 1 Pharmacy Department, Hospital Universitario Donostia, 20014 Donostia, Spain; 2 Pharmacy Department, Hospital de Urduliz Alfredo Espinosa, 48610 Vizcaya, Spain; 3 Pharmacy Department, Hospital HLA Vistahermosa, 03015 Alicante, Spain; 4 Pharmacy Department, Hospital Universitario Virgen del Rocío, 41013 Sevilla, Spain; 5 Pharmacy Department, Hospital General Universitario Gregorio Marañón, Instituto de Investigación Sanitaria Gregorio Marañón (IiSGM), 28007 Madrid, Spain; 6 Pharmacy Department, Hospital de Mataró, 08304 Barcelona, Spain; 7 Pharmacy Department, Hospital de Cabueñes, 33394 Asturias, Spain; 8 Pharmacy Department, Hospital Universitario Ntra. Sra. de Candelaria, 38010 Tenerife, Spain; 9 Pharmacy Department, Hospital Universitario Sta. Lucía, 30302 Cartagena, Spain; 10 Pharmacy Department, Complejo Hospitalario Torrecárdenas, 04009 Almería, Spain; 11 Pharmacy Department, Hospital Universitario Rio Hortega, 47012 Valladolid, Spain; 12 Pharmacy Department, Complejo Hospitalario de Jaén, 23009 Jaén, Spain; 13 Pharmacy Department, Hospital El Bierzo, 24411 León, Spain

**Keywords:** malnutrition, cancer, nutrition screening tools, ambulatory oncology, clinical nutrition, malnutrition diagnosis

## Abstract

**Background/Objectives**: Disease-related malnutrition is highly prevalent in oncology and is associated with poor clinical outcomes. Early detection through nutritional screening is essential; however, the optimal screening tool for ambulatory cancer patients remains uncertain. This study aimed to evaluate the agreement and diagnostic performance of the malnutrition screening tool (MST) and NUTRISCORE compared with the Global Leadership Initiative on Malnutrition (GLIM) criteria in a large nationwide cohort of ambulatory cancer patients. **Methods**: In this multicenter, observational, cross-sectional nationwide study, adult patients attending oncology day hospitals for intravenous antineoplastic treatment between April and November 2021 were included. Nutritional risk was assessed using MST (cut-off ≥ 2) and NUTRISCORE (cut-off ≥ 5). Malnutrition was diagnosed according to GLIM criteria. Agreement between tools was assessed with Cohen’s kappa, and diagnostic performance was evaluated by sensitivity, specificity, accuracy, positive predictive value, and negative predictive value. Analyses were stratified by tumor nutritional risk and cancer stage. **Results**: Among 4440 patients from 86 hospitals, 50.7% met the GLIM criteria for malnutrition; 72.5% had moderate and 27.5% severe malnutrition. MST identified 37.5% of patients as being at nutritional risk, compared with 17.3% identified by NUTRISCORE. Agreement between MST and NUTRISCORE was moderate overall (κ = 0.48; 95% CI, 0.45–0.51), but varied markedly according to tumor nutritional risk, ranging from high agreement in high-risk tumors (κ = 0.82) to low agreement in low-risk tumors (κ = 0.28). Relative to GLIM, MST was more sensitive than NUTRISCORE (0.51 vs. 0.27), whereas NUTRISCORE was more specific (0.92 vs. 0.76) and had a higher positive predictive value (0.77 vs. 0.68). Negative predictive value was low for both tools. **Conclusions**: GLIM-defined malnutrition was highly prevalent in this large cohort of ambulatory patients with cancer. MST provided greater case detection, whereas NUTRISCORE showed a more conservative profile with higher specificity but substantially lower sensitivity. These findings suggest that the choice of screening tool should consider clinical context- and tumor-related nutritional risk, and that neither instrument alone reliably excludes malnutrition in outpatient oncology settings.

## 1. Introduction

Disease-related malnutrition in patients with cancer represents a major clinical challenge, with a reported prevalence ranging between 30% and 70% depending on tumor location and stage [1]. Malnutrition in this population is consistently associated with increased morbidity and mortality, impaired quality of life, reduced functional capacity, higher rates of treatment-related toxicity, poorer tolerance to chemotherapy and radiotherapy, increased postoperative complications, and overall worse oncologic outcomes [2].

In oncology, malnutrition is a dynamic and progressive condition. It frequently develops early in the disease course and may worsen as the tumor burden increases and systemic inflammation intensifies. If not identified and treated in a timely manner, disease-related malnutrition may progress to cancer cachexia, a multifactorial syndrome characterized by ongoing loss of skeletal muscle mass that is only partially reversible with conventional nutritional support [3].

Given the high incidence of malnutrition in oncology, its progressive nature, and its profound clinical consequences, cancer patients should be systematically screened for nutritional risk throughout the entire cancer process, in order to implement nutritional strategies in a timely manner to prevent further deterioration and avoid progression to irreversible stages.

Several tools are currently used for nutritional screening in oncology populations. The ESPEN clinical guidelines recommend the malnutrition screening tool (MST) and the Malnutrition Universal Screening Tool (MUST) for patients with cancer [4], whereas NUTRISCORE was specifically developed for use in ambulatory oncology settings [5]. Although multiple studies have compared instruments such as NRS-2002, MUST, MST, and NUTRISCORE in this population, there is still no clear consensus regarding their relative diagnostic performance [6,7,8,9,10]. Moreover, there is insufficient evidence to support the superiority of one tool over another, as results vary depending on the reference standard used (e.g., GLIM criteria or PG-SGA), the clinical setting (outpatient, inpatient, or surgical), and the oncologic population studied [11,12].

Previous studies assessing the diagnostic performance of MST and NUTRISCORE have used PG-SGA [13,14] or GLIM criteria [15,16,17] as reference standards, reporting heterogeneous results influenced by care setting and tumor type. Importantly, studies comparing MST and NUTRISCORE against GLIM have largely been conducted in hospitalized patients or in specific tumor sites, such as lung, head and neck, or gastrointestinal cancers. The variability observed in ambulatory oncology populations highlights the need for further research in this context. To our knowledge, no study has comprehensively evaluated the performance of MST and NUTRISCORE against the GLIM diagnostic criteria in a broad cohort of ambulatory cancer patients encompassing multiple tumor locations. Thus, the main objective of this study was to analyze the agreement between MST and NUTRISCORE and their diagnostic performance against GLIM criteria.

## 2. Materials and Methods

### 2.1. Study Design and Participants

This was a multicenter, observational, cross-sectional, nationwide study in standard clinical practice. Patients were recruited and evaluated upon admission to the oncohematology day hospital to receive intravenous antineoplastic treatment. Recruitment took place between April and November 2021.

To ensure random selection, a systematic sampling method was used: patients with even-numbered medical records were recruited on even-numbered days, while those with odd-numbered medical records were recruited on odd-numbered days.

We considered the following exclusion criteria: age under 18 years, refusal to participate, or the presence of cognitive impairment. All patients included in the study signed an informed consent form prior to participation.

The OncoNutridos study was promoted by the Spanish Foundation of Hospital Pharmacy and was carried out with the collaboration of the clinical nutrition group and the group for the development of oncological pharmacy of the Spanish Society of Hospital Pharmacy. The study was approved by the Clinical Research Ethics Committee of the Basque Country (CEIm-E), as well as by the ethics committees of all participating hospitals (reference code: OncoNutridos, date of approval: 25 March 2020).

### 2.2. Demographic, Clinical, and Anthropometric Measurements

Demographic characteristics, clinical variables, and anthropometric data were collected at the time of the patient’s admission to the chemotherapy suite. Trained healthcare professionals, including pharmacists, nurses, and dieticians, interviewed all patients.

Anthropometric data included weight (kg), height (m), both obtained from the patient’s medical record, and calf circumference (CC). The most recent weight, used for calculating anticancer drug dosages, was recorded. Usual weight was obtained through the patient’s interview, which allowed us to assess and quantify the weight loss over time (3–6 months or >6 months). Body mass index (BMI) was calculated as weight in kilograms divided by height in meters squared (kg/m^2^).

Tumor location, TNM stage (I–II, III–IV), and type of cancer treatment (chemotherapy, radiotherapy, or oncologic surgery) were obtained from the patient’s medical records. Tumor locations were classified into three categories based on NUTRISCORE classification: high-, medium-, and low-nutritional-risk tumors. This classification is based on previous evidence showing that the risk and prevalence of malnutrition vary significantly according to tumor location, with cancers affecting the upper gastrointestinal tract and head and neck being associated with higher nutritional risk, while others, such as breast or prostate cancer, are generally considered lower risk [5,18]. This classification is clinically meaningful, as it allows for a more tailored identification of patients at nutritional risk, taking into account disease-specific factors.

### 2.3. Assessment of Malnutrition Risk and Nutritional Status

Two different screening tests were used to identify the risk of malnutrition: malnutrition screening tool (MST) [19], with a cut-off point of ≥2, and NUTRISCORE, which includes the MST and adds tumor location and treatment, setting the cut-off point at score ≥ 5.

Diagnosis of malnutrition was performed using the GLIM criteria as a reference in all patients, and all patients underwent both screening tests [20,21,22]. These criteria require at least one phenotypic criterion (weight loss, low body mass index, and/or reduced muscle mass) and one etiological criterion (decreased intake, a chronic gastrointestinal condition, or systemic inflammation). Severity of malnutrition was graded according to the cut-off points suggested for each phenotypic criterion.

Reduced muscle mass was assessed via calf circumference (CC), measured at the widest point using a non-extendable tape measure, with the patient seated and feet flat on the floor. The raw CC was adjusted for the patient’s BMI as follows: if BMI was <18 kg/m^2^ when adding 4 cm, if BMI was 25–29.9 kg/m^2^ when subtracting 3 cm, and if BMI was 30–39.9 kg/m^2^ or >40 kg/m^2^ when subtracting 7 cm and 12 cm, respectively. Cut-off values for reduced muscle mass were 33 cm in men and 32 cm in women [21].

We also estimated Appendicular Skeletal Mass (ASM) using the validated equation [23]: ASM (kg) = −10 + (CC × 0.768) − (age × 0.029) + (sex × 7.523) (sex: men = 1; women = 2; all assumed with), and calculated the Appendicular Skeletal Mass Index (ASMI) as ASM/height^2^.

With respect to the etiological criteria, all participants were considered to have moderate disease-related inflammation, fulfilling this requirement based on their diagnosis of active cancer and current receipt of intravenous antineoplastic treatment [22]. A decreased intake was considered when the patient presented a reduction of at least 50% of the baseline amount for at least one week, or any reduction for at least two weeks.

All patients at risk of DRM received general dietary recommendations provided within the study protocol, aimed at increasing caloric and protein intake. These recommendations were delivered to patients by the study investigators, who could include nutrition support pharmacists, nutritionists, dietitians, or physicians, depending on the resources available at each participating hospital. No standardized follow-up regarding adherence to these recommendations or their impact on nutritional status was performed.

### 2.4. Statistical Analysis

Descriptive statistics are presented as mean ± standard deviation (SD) for continuous variables and as absolute (n) and relative (%) frequencies for categorical variables. Malnutrition, assessed by the GLIM criteria, was considered the reference and compared to the other nutritional screening tools. Agreement between these tests and GLIM criteria was analyzed by the kappa (k) statistic. Diagnostic capabilities of the screening tests were evaluated by different metrics: sensitivity, specificity, accuracy, predictive positive value (PPV), and negative predictive value (NPV), using standard formulae. No formal adjustment for multiple comparisons was applied, as subgroup analyses by tumor type were considered exploratory. All analyses were conducted in R version 4.4.2 (R Core Team 2025).

## 3. Results

### 3.1. Patient Characteristics, Risk of Malnutrition, and Malnutrition Prevalence

A total of 4548 patients were included from 86 hospitals between April and November 2021. Of these, 108 patients were excluded due to missing data, resulting in a final sample of 4440 patients (57% women). Low-risk nutritional tumors were the most prevalent (63.6%), with breast and colorectal tumors being the main locations (23.9% and 18.5%, respectively). Most patients (72%) were at an advanced tumor stage (stage III–IV). Regarding treatment, most of the participants were receiving chemotherapy alone (63%), and 25% were oncology surgery patients (Table 1).

Overall, 51.9% of patients had experienced weight loss, with a median loss of 7.1 kg [0.2–74.0]. The prevalence of weight loss increased according to the nutritional risk associated with tumor location, affecting 48.2% of patients with low-risk tumors, 51.6% of those with medium-risk tumors, and 68.6% of those with high-risk tumors. Median weight loss was 8.6 kg [0.5–74.0], 7.1 kg [0.5–73.5], and 8.6 kg [1.0–40.0], respectively.

The mean score for MST was 1.25 ± 1.40 (SD) [range: 0–5]; for NUTRISCORE, the mean value was 2.79 ± 1.78 (SD) [range: 0–9]. Globally, using MST as a screening tool, 37.48% of patients were classified as at risk of malnutrition, whereas NUTRISCORE detected 17.34% of patients as at nutritional risk. The detection of risk of malnutrition fell in both tests in accordance with the nutritional risk associated with tumor location (Table 2).

The weight loss matched the GLIM diagnostic criteria in 24.4% of patients. Reduced muscle mass was present at 29.98% of patients, and low BMI was detected in 11.8% of patients. Reduced intake or absorption was detected in 27.5% of patients. All patients fulfilled the inflammation criterion, as they were undergoing active chemotherapy for cancer. According to the GLIM diagnostic criteria, 2253 patients (50.74%) presented malnutrition, of whom 72.52% was moderate (1634) and severe in 27.48% (619). Malnutrition prevalence changed depending on tumor location (Table 2), ranging from 37.7% in breast cancer to 75.0% in patients with gastric tumors.

### 3.2. Agreement Between MST and NUTRISCORE

The overall agreement between MST and NUTRISCORE was moderate, with an observed agreement of 78.0% and a kappa coefficient of 0.48, indicating a moderate level of concordance between the two risk classification tools (Table 3).

To assess whether agreement between both screening tools varied according to tumor location, concordance was analyzed in relation to the nutritional risk associated with each tumor type. Overall, agreement between MST and NUTRISCORE progressively decreased as the nutritional risk of the tumor declined (Table 4). Agreement was very high in high-risk tumors (κ = 0.81), indicating that both tools classified patients in a highly consistent manner. In medium-risk tumors, agreement was moderate (κ = 0.58), with the highest levels observed in lung and biliary tract cancers (κ = 0.62 and 0.64, respectively). In contrast, agreement between MST and NUTRISCORE was clearly limited in low nutritional risk tumors, with κ values generally < 0.35 across all locations. The highest agreement in this group was observed in central nervous system (CNS) and prostate tumors; however, these estimates were associated with substantial uncertainty due to the small number of cases.

### 3.3. Diagnostic Capabilities of the Screening Tests

When GLIM criteria were used as the diagnostic reference, clear differences emerged in the performance of MST and NUTRISCORE (Table 5). MST (≥2) identified approximately half of the patients classified as malnourished (sensitivity 0.51), whereas NUTRISCORE (≥5) detected only one quarter of cases (sensitivity 0.27), highlighting the limited ability of both instruments to achieve complete detection of malnutrition. However, NUTRISCORE demonstrated a greater capacity to correctly classify patients as “not at risk” (specificity 0.92 vs. 0.76). Both tools showed low negative predictive values, indicating that a substantial proportion of patients categorized as “not at risk” could still be malnourished; thus, a negative screening result cannot reliably exclude malnutrition.

When the cohort was stratified according to the nutritional risk associated with tumor location, meaningful discrepancies arose in the diagnostic accuracy of MST and NUTRISCORE against the GLIM criteria (Table 6). In tumors classified as high nutritional risk, both tools identified a similar proportion of patients as being at risk (54.6% with MST and 51.5% with NUTRISCORE), with comparable sensitivities and positive predictive values. However, this pattern did not persist in tumors of medium and low nutritional risk. In these groups, MST identified substantially more patients at risk and demonstrated consistently higher sensitivity, whereas NUTRISCORE showed markedly lower sensitivity. Conversely, NUTRISCORE exhibited systematically higher specificity, particularly in medium- and low-risk tumors (0.89 and 0.97, respectively), resulting in higher positive predictive values in these groups. Negative predictive values were modest for both tools across all categories. Overall, MST provided greater case detection across risk strata, while NUTRISCORE maintained a more conservative profile characterized by higher specificity, especially in tumors with lower baseline nutritional risk.

Metrics related to the diagnostic performance of MST and NUTRISCORE by specific tumor location are shown in Supplementary Materials (Tables S1–S3). Broadly, both screening tools showed the highest sensitivity (≈60%) in head and neck, esophagus, gastric, and pancreatic tumors, all of them classified as high-risk nutritional tumors. In the rest of the cancer locations analyzed, MST had consistently higher sensitivities than NUTRISCORE. MST also showed a good performance (≈50%) in lung and biliary tract tumors (among medium-risk nutritional locations), and in colorectal and leukemia cancers in low-risk nutritional locations. In tumors classified as medium- and low-nutritional-risk, NUTRISCORE showed low sensitivities (<0.44 and <0.25, respectively), indicating a limited ability to identify malnutrition according to the GLIM diagnostic criteria. In contrast, NUTRISCORE specificity was consistently high, exceeding 0.80 across all tumor types and reaching values close to 1.00 in some tumors. Overall, accuracy was moderate across most tumor types, with comparable values between both methods and slight advantages observed for MST in several tumor locations.

We also split the cohort to determine if the agreement and performance of both screening tools were influenced by cancer stage. The agreement between MST and NUTRISCORE was moderate in both cancer stages. The kappa index in stages I-II was 0.40 (IC95%: 0.33–0.47), while in stages III–IV it was slightly higher (κ = 0.50; IC95%: 0.46–0.53). When analyzing performance, MST demonstrated higher sensitivities than NUTRISCORE in both stages I–II (0.43 vs. 0.18; CI95% [0.39–0.47] and [0.15–0.21], respectively) and stages III–IV (0.53 vs. 0.29; CI95% [0.51–0.55] and [0.27–0.31], respectively). In contrast, NUTRISCORE showed higher specificities in both groups (0.96 and 0.90, CI95% [0.95–0.98] and [0.89–0.92] respectively), as well as higher positive predictive values. The negative predictive value was greater with MST in both stages, while overall accuracy was similar between tools in stages I–II (≈0.65) and slightly higher for MST in stages III–IV (0.63 vs. 0.57).

## 4. Discussion

The present multicenter study provides a comparison of two widely used nutritional screening tools in ambulatory oncology patients and evaluates their diagnostic performance against malnutrition defined according to the GLIM criteria. Several relevant findings emerge from this analysis.

First, our results confirm the high prevalence of disease-related malnutrition in ambulatory oncology populations. In this cohort, more than half of the patients (50.7%) were classified as malnourished according to GLIM criteria. This finding is consistent with previous studies reporting a substantial burden of malnutrition among oncology patients and reinforces the clinical importance of systematic nutritional assessment in cancer care [24,25]. Notably, this prevalence was observed despite the predominance of tumor types traditionally considered to have lower nutritional risk, such as breast and colorectal cancer. These findings support the concept that malnutrition is not limited to tumors classically associated with severe nutritional impairment but may affect a wide spectrum of oncology patients [1].

Second, differences were observed in the diagnostic performance of the screening tools. Overall, MST identified a substantially higher proportion of patients at nutritional risk than NUTRISCORE (37.5% vs. 17.3%). Other authors [15] reported higher detection rates (52% MST vs. 38% NUTRISCORE) when evaluating screening performance across tumor sites such as head and neck, digestive, and colorectal cancers. The higher prevalence observed in their cohort likely reflects a study population enriched with tumors associated with higher nutritional risk. Another contributing factor to these differences is the sample size, as the number of patients recruited by Gascón-Ruiz differs significantly from our own cohort. In addition, involuntary weight loss was reported in 60.6% of patients in their study compared with 51.9% in our cohort. Despite differences in absolute prevalence, both studies show a consistent pattern in which MST identifies a greater proportion of patients at nutritional risk than NUTRISCORE, suggesting higher sensitivity of MST in heterogeneous oncology populations.

Third, the moderate agreement observed between both screening tools (κ = 0.48) indicates that they identify partially different patient subsets. Concordance varied considerably according to tumor location and associated nutritional risk. In tumors traditionally associated with severe nutritional impairment—such as upper gastrointestinal or pancreatic cancers—agreement was very high. In contrast, concordance progressively decreased in tumors with lower baseline nutritional risk. This pattern likely reflects the design of NUTRISCORE, which incorporates tumor location and oncologic treatment into its scoring system. Accordingly, the clinical utility of NUTRISCORE appears strongly dependent on the tumor’s nutritional impact. In high-risk tumors, the tool demonstrated excellent agreement (κ = 0.81), higher than the κ = 0.63 reported by Gascón-Ruiz. However, agreement decreased substantially in our overall sample (κ = 0.48) due to the relatively low prevalence of these tumors (14.6%).

This finding suggests that NUTRISCORE may underestimate nutritional risk in tumors with low nutritional impact. Because these tumor locations do not contribute points to the scale, patients must experience greater weight loss to reach the diagnostic threshold of ≥5 points.

Consequently, our results suggest that both tools may be interchangeable in tumors with high nutritional risk and are consistent with results previously published by other authors [15]. In medium-risk tumors, agreement was lower (κ = 0.57) and varied according to tumor location. Higher concordance was observed in lung and biliary tract cancers, whereas lower agreement in ovarian cancer indicates greater discrepancies between the instruments. In tumors with low nutritional risk, the tools cannot be considered interchangeable. These findings indicate that the interchangeability of screening instruments is highly dependent on the clinical context.

In advanced stages (III–IV), MST demonstrated higher global accuracy compared to NUTRISCORE (0.63 vs. 0.57), suggesting that MST remains more robust in identifying nutritional risk as the disease progresses. In contrast, NUTRISCORE may lose some of its screening effectiveness in these more complex stages. Despite this difference in individual performance, the degree of agreement between both tools remained moderate and even increased slightly in advanced stages, as reflected by the Kappa index (0.50 vs. 0.40).

Fourth, regarding diagnostic performance compared with GLIM criteria, MST showed approximately double the sensitivity of NUTRISCORE (50.7% vs. 26.5%), indicating greater ability to detect malnourished patients. However, MST still failed to identify approximately 49% of cases. In contrast, NUTRISCORE demonstrated higher specificity (92% vs. 76%), suggesting greater reliability for ruling out malnutrition when patients are classified as having “no risk”.

Both screening methods showed good positive predictive value (PPV), although NUTRISCORE presented a higher PPV (77%), indicating that a positive result is relatively reliable. Conversely, both tools showed low negative predictive values (NPVs), particularly NUTRISCORE (55%), indicating that a negative screening result does not reliably exclude malnutrition.

These findings highlight important limitations in the screening capacity of both tools when compared with GLIM criteria. Although MST is twice as sensitive as NUTRISCORE, it still misses nearly half of malnourished patients. This limitation is even more pronounced for NUTRISCORE, which fails to identify approximately three out of four malnourished patients despite its high specificity and strong confirmatory value for positive results. Together, these results support the need to complement nutritional screening with structured diagnostic assessment, particularly in oncology populations at higher risk of malnutrition. From a practical perspective, a stepwise approach could be considered, using the MST—due to its higher sensitivity—as an initial screening tool, followed by a comprehensive nutritional assessment in patients identified as being at risk or whenever clinical suspicion persists despite a negative result (particularly in tumors with low-to-medium nutritional risk, given the observed low sensitivity). This strategy may help minimize false negatives while ensuring the timely identification and management of malnutrition in routine clinical practice.

The lower sensitivity observed in our study compared with the findings of other authors may be explained by differences in tumor distribution. In the Gascón-Ruiz cohort, more than 50% of patients had high-risk tumors (head and neck or upper gastrointestinal), whereas these represented only 14.6% of our sample. Because NUTRISCORE assigns points based on tumor location, patients in their study were more likely to reach the ≥5 score threshold. In our cohort, dominated by tumors with lower nutritional impact, the tool relied mainly on severe weight loss to generate a positive result, which likely explains the lower sensitivity observed (26.5%). However, other clinical factors may also have contributed to this discrepancy. In particular, variations in treatment intensity could play a relevant role, as NUTRISCORE incorporates oncologic treatment as part of its scoring system, assigning higher scores to more aggressive regimens such as concomitant radiochemotherapy. As a result, this factor could not be fully explored in our analysis and may have contributed to the observed differences in sensitivity between studies. These factors should be considered when interpreting differences in diagnostic performance across studies.

According to this, our results suggest that in populations where tumors with lower nutritional impact predominate, NUTRISCORE loses the additional scoring contribution derived from tumor location and treatment intensity. Consequently, the tool shows a high rate of false negatives and reduced sensitivity, even when the prevalence of malnutrition remains high.

Based on these discrepancies, our findings suggest that a re-evaluation of the NUTRISCORE scoring criteria may be warranted to improve its sensitivity in contemporary oncological settings. First, our data indicate the need for a re-assignment of the nutritional risk attributed to certain malignancies currently classified as low or moderate risk. In our cohort, these tumors exhibited a high prevalence of malnutrition according to GLIM criteria, but failed to trigger the NUTRISCORE threshold because of their low baseline score. Second, the current model might benefit from incorporating age > 70 years as a specific risk factor. This is supported by findings from the OncoNutridos study [1], where the prevalence of malnutrition reached 62.86% in patients over 70 years, regardless of tumor location, compared to 50.7% in younger patients. These proposed modifications, including the incorporation of age > 70 years as a risk factor, should be considered hypothesis-generating and require validation in prospective studies specifically designed to assess their impact on the diagnostic performance of NUTRISCORE.

## 5. Conclusions

In the OncoNutridos study, the prevalence of malnutrition according to the Global Leadership Initiative on Malnutrition (GLIM) criteria was high (50.7%), affecting one out of every two oncology patients undergoing active treatment, with a predominance of moderate forms (72.5%). This confirms that malnutrition remains a clinical issue of great magnitude in oncology.

Nutritional screening showed relevant differences between tools. MST (≥2) identified a higher number of patients at risk and presented higher overall sensitivity, while NUTRISCORE (≥5) was more specific but had low sensitivity. Consequently, NUTRISCORE adopts a more conservative profile, while MST offers greater detection capacity.

The degree of agreement between MST and NUTRISCORE was moderate, and increased slightly in advanced stages (III–IV). However, MST proved to be a more robust screening tool across all stages, particularly due to its higher sensitivity in advanced disease compared to NUTRISCORE.

Overall, MST appears more suitable as an initial detection tool due to its higher sensitivity, while NUTRISCORE could be useful in contexts where specificity is prioritized. However, the high prevalence of malnutrition supports the need for systematic nutritional assessment in oncology.

Neither tool showed a high negative predictive value, indicating that a negative screening result does not rule out malnutrition according to GLIM.

### Strengths and Limitations

This study has several strengths that should be considered when interpreting the results. First, it includes a large multicenter cohort of ambulatory oncology patients recruited from 86 hospitals, providing a broad and heterogeneous representation of real-world clinical practice. The large sample size allowed the inclusion of a wide range of tumor locations and nutritional risk profiles, enabling subgroup analyses according to tumor nutritional risk and stage. This aspect is particularly relevant because the performance of nutritional screening tools may vary depending on tumor characteristics and disease severity.

Second, the study evaluates the performance of two widely used nutritional screening tools against the GLIM criteria, which currently represent an internationally accepted framework for the diagnosis of malnutrition. By comparing screening tools with standardized diagnostic criteria, our study provides clinically meaningful information regarding their ability to identify patients who may require further nutritional assessment.

Third, the multicenter design and inclusion of patients undergoing different oncologic treatments enhance the external validity of the findings. This design reflects the heterogeneity typically observed in ambulatory oncology populations and therefore increases the applicability of the results to routine clinical practice.

However, several limitations should also be acknowledged.

First, although GLIM criteria were used as the diagnostic reference for malnutrition, it should be noted that GLIM itself requires the use of a screening tool as the first step in the diagnostic process. Consequently, the choice of screening instrument may influence the identification of patients entering the diagnostic pathway, which could partially affect comparisons between screening methods.

Second, some tumor subgroups included a relatively small number of patients, which may have limited the precision of diagnostic performance estimates in specific cancer locations. This limitation was particularly relevant in tumors with low incidence within the cohort.

Third, the assessment of inflammation in this study was based on the “active disease” criterion. According to the Guidance for assessment of the inflammation etiologic criterion for GLIM diagnosis of malnutrition [22], the presence of an acute or chronic disease, infection, or injury typically associated with inflammatory activity is sufficient to meet this etiologic requirement. Although measuring C-reactive protein (CRP) is recommended to confirm inflammation in ambiguous cases, clinical judgment grounded in the underlying diagnosis remains a fundamental pillar of the clinician’s assessment. In this study, given the observational, real-world nature of the data, systemic CRP measurement was not performed in all patients; therefore, the presence of disease-related inflammation was primarily determined based on the clinical diagnosis and the ongoing administration of antineoplastic treatment.

Fourth, muscle mass assessment represents an important methodological consideration in our study. Although calf circumference (CC) is a practical and widely applicable surrogate, its accuracy compared with gold-standard techniques such as CT-based measurements remains variable. Previous studies in oncology populations have reported a relatively low sensitivity of CC for detecting low muscle mass [26], suggesting a potential underestimation when compared to CT-derived measures, while others have shown good agreement, with high sensitivity and specificity [27]. These discrepancies highlight the inherent limitations of CC as a proxy for muscle mass. Nevertheless, given its non-invasive, rapid, and low-cost nature, CC remains a feasible and pragmatic option in large-scale, real-world studies such as ours, which involved 86 hospitals and a large cohort of patients. This trade-off between feasibility and precision should be considered when interpreting our findings.

Finally, the study was conducted in ambulatory oncology settings, and therefore, the findings may not be directly generalizable to hospitalized cancer patients or to populations with different clinical characteristics.

Despite these limitations, the large sample size and multicenter design provide robust evidence regarding the comparative performance of MST and NUTRISCORE as nutritional screening tools in ambulatory oncology populations.

## Figures and Tables

**Table 1:** Demographic- and tumor-related clinical variables.

Variables	n (%)	Variables	n (%)
*Sex*		*Tumor location*	
Male	1909 (43.0)	*High-risk nutritional tumors (n = 649)*	
Female	2531 (57.0)	Head and neck	104 (2.3)
		Esophagus	67 (1.5)
*Age (years)*	61.1 [53.0; 74.0]	Stomach	164 (3.7)
		Small intestine	34 (0.8)
*Cancer stage*		Digestive lymphomas	47 (1.1)
I–II	1228 (27.7)	Pancreas	233 (5.2)
III–IV	3212 (72.3)	*Medium-risk nutritional tumors (n = 967)*	
		Lung	554 (12.5)
*BMI* (Kg/m^2^)		Ovarian	201 (4.5)
Mean (SD)	25.9 (4.9)	Endometrium	67 (1.5)
Median	25.3	Biliary tract	54 (1.2)
		Liver	48 (1.1)
*BMI by groups* (Kg/m^2^)		Kidney	43 (1.0)
<18.5	162 (3.7)	*Low-risk nutritional tumors (n = 2824)*	
18.5–19.9	227 (5.1)	Breast	1061 (23.9)
20.0–24.9	1676 (37.7)	Colorectal	823 (18.5)
24.9–29.9	1563 (35.2)	Leukemias	91 (2.0)
≥30	812 (18.3)	Other lymphomas	285 (6.4)
		Prostate	70 (1.6)
*ASMI* (Kg/m^2^) *by sex*		Bladder	99 (2.2)
Male (Mean; SD)	7.28 (0.84)	CNS	20 (0.5)
Female (Mean; SD)	5.25 (0.98)	Other tumors	375 (8.4)

Data are shown as median [IQR] for continuous variables and number of cases (%) for categorical variables.

**Table 2:** Risk of malnutrition and malnutrition prevalence by tumor location.

Tumor Location	Risk of Malnutrition		Malnutrition
	MST	NUTRISCORE	
	n (%)	n (%)	n (%)
High-risk nutritional tumors	355 (54.7)	335 (51.6)	438 (67.49)
Head and neck	48 (46.2)	54 (51.9)	64 (61.5)
Esophagus	43 (64.2)	40 (59.7)	44 (65.7)
Stomach	100 (61.0)	92 (56.1)	123 (75.0)
Small intestine	13 (38.2)	8 (23.5)	18 (52.9)
Digestive lymphomas	15 (31.9)	14 (29.8)	23 (48.9)
Pancreas	136 (58.4)	127 (54.5)	166 (71.2)
Medium-risk nutritional tumors	378 (39.1)	210 (21.7)	505 (52.22)
Lung	228 (41.2)	140 (25.3)	294 (53.1)
Ovary	74 (36.8)	24 (11.9)	104 (51.7)
Endometrium	22 (32.8)	13 (19.4)	30 (44.8)
Biliary tract	25 (46.3)	15 (27.8)	27 (50.0)
Liver	14 (29.2)	12 (25.0)	25 (52.1)
Kidney	15 (34.9)	6 (14.0)	25 (58.1)
Low-risk nutritional tumors	931 (33.0)	225 (8.0)	1310 (46.39)
Breast	280 (26.4)	39 (3.7)	400 (37.7)
Colorectal	337 (31.8)	103 (12.5)	458 (55.7)
Leukemias	34 (37.4)	8 (8.8)	45 (49.5)
Other lymphoma	89 (31.2)	23 (8.1)	135 (47.4)
Prostate	20 (28.6)	7 (10.0)	35 (50.0)
Bladder	31 (31.3)	8 (8.1)	54 (54.5)
CNS	5 (25.0)	2 (10.0)	4 (20.0)
Other tumors	135 (36.0)	35 (13.2)	179 (47.7)

MST: malnutrition screening tool.

**Table 3:** Agreement between MST and NUTRISCORE.

Variable	Value
Observed agreement (po)	0.780
Expected agreement (pe)	0.660
Cohen’s kappa (κ)	0.481
Standard error	0.0148
95% CI for κ	0.452–0.510
Interpretation	Moderate agreement

**Table 4:** Agreement between MST and NUTRISCORE regarding tumor location.

Tumor Location	Kappa (κ)	95% CI	Standard Error
High nutritional risk	0.82	0.77–0.86	0.02
Head and neck	0.73	0.60–0.86	0.07
Esophagus	0.84	0.70–0.97	0.07
Stomach	0.87	0.80–0.95	0.04
Small intestine	0.66	0.39–0.94	0.14
Digestive lymphomas	0.85	0.69–1.01	0.08
Pancreas	0.80	0.72–0.88	0.04
Medium nutritional risk	0.58	0.52–0.63	0.03
Lung	0.64	0.58–0.71	0.03
Ovary	0.38	0.23–0.53	0.08
Endometrium	0.51	0.27–0.75	0.12
Biliary tract	0.62	0.40–0.83	0.11
Liver	0.58	0.31–0.84	0.14
Kidney	0.46	0.15–0.77	0.16
Low nutritional risk	0.28	0.24–0.33	0.02
Breast	0.18	0.09–0.27	0.05
Colorectal	0.32	0.25–0.40	0.04
Leukemias	0.27	0.04–0.51	0.12
Other lymphoma	0.30	0.16–0.45	0.07
Prostate	0.35	0.06–0.64	0.15
Bladder	0.32	0.08–0.57	0.12
CNS	0.50	−0.03–1.02	0.27
Other tumors	0.29	0.17–0.40	0.07

**Table 5:** Diagnostic performance of MST and NUTRISCORE compared with GLIM.

	Score	n (%) Positive	Accuracy (CI95%)	Sensitivity (CI95%)	Specificity (CI95%)	PPV (CI95%)	NPV (CI95%)
MST	≥2	1664 (37.5)	0.63 (0.62–0.65)	0.51 (0.49–0.53)	0.76 (0.74–0.78)	0.68 (0.66–0.71)	0.60 (0.58–0.62)
NUTRISCORE	≥5	770 (17.3)	0.58 (0.57–0.60)	0.27 (0.25–0.28)	0.92 (0.91–0.93)	0.77 (0.75–0.80)	0.55 (0.53–0.57)

MST: malnutrition screening tool; PPV: positive predictive value; NPV: negative predictive value.

**Table 6:** Diagnostic performance of MST and NUTRISCORE by tumor nutritional risk.

Group		n (%) Positive	Sensitivity (CI95%)	Specificity (CI95%)	PPV (CI95%)	NPV (CI95%)	Accuracy (CI95%)
**High risk**	MST	355 (54.6)	0.61 (0.57–0.66)	0.59 (0.53–0.66)	0.76 (0.71–0.80)	0.43 (0.37–0.49)	0.61 (0.57–0.65)
	NUTRISCORE	335 (51.5)	0.58 (0.53–0.62)	0.61 (0.54–0.68)	0.75 (0.71–0.80)	0.41 (0.34–0.47)	0.59 (0.55–0.62)
**Medium risk**	MST	378 (39.1)	0.52 (0.48–0.56)	0.75 (0.71–0.79)	0.69 (0.65–0.74)	0.59 (0.55–0.63)	0.63 (0.60–0.66)
	NUTRISCORE	210 (21.7)	0.32 (0.28–0.36)	0.89 (0.86–0.92)	0.76 (0.70–0.82)	0.54 (0.51–0.58)	0.59 (0.56–0.62)
**Low risk**	MST	931 (33.0)	0.47 (0.44–0.49)	0.79 (0.77–0.81)	0.66 (0.63–0.69)	0.63 (0.61–0.65)	0.64 (0.62–0.66)
	NUTRISCORE	225 (7.9%)	0.14 (0.12–0.16)	0.97 (0.97–0.98)	0.82 (0.77–0.87)	0.57 (0.55–0.59)	0.59 (0.57–0.61)

MST: malnutrition screening tool; PPV: positive predictive value; NPV: negative predictive value.

**Figure A1:**
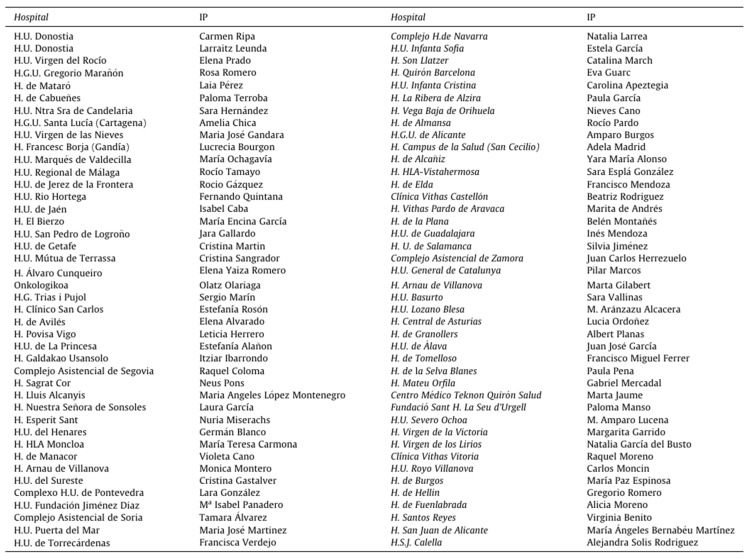
Hospitals enrolled and IP.

## Data Availability

The original contributions presented in this study are included in the article/Supplementary Materials. Further inquiries can be directed to the corresponding author.

## Supplementary Materials

The following supporting information can be downloaded at: https://www.mdpi.com/article/10.3390/nu18091452/s1, Table S1: MST and NUTRISCORE diagnostic capabilities based on tumor location: high nutritional risk tumors; Table S2: MST and NUTRISCORE diagnostic capabilities based on tumor location: medium nutritional risk tumors; Table S3: MST and NUTRISCORE diagnostic capabilities based on tumor location: low nutritional risk tumors.
