# Supplementary material for: Diagnostic Performance and Agreement of MST and NUTRISCORE Compared with GLIM Criteria in Ambulatory Cancer Patients: Results from the OncoNutridos Study
Source: Nutrients. 2026 Apr 30;18(9):1452. doi: 10.3390/nu18091452 (PMC13165140; doi:10.3390/nu18091452)
Supplement: Supplementary file 1 [file nutrients-18-01452-s001.zip › nutrients-4275650-supplementary.pdf]

SUPPLEMENTARY MATERIAL

Table S1. MST and NUTRISCORE diagnostic capabilities based on tumor location: High nutritional risk tumors.

Tumor location	Tool	Sensitivity (CI95%)	Specificity (CI95%)	PPV (CI95%)	NPV (CI95%)	Accuracy (CI95%)
High nutritional risk						
Head and neck	MST	0.61 (0.49-0.73)	0.78 (0.65-0.90)	0.81 (0.70-0.92)	0.55 (0.43-0.68)	0.67 (0.58-0.76)
	NUTRISCORE	0.66 (0.54-0.77)	0.70 (0.56-0.84)	0.78 (0.66-0.89)	0.56 (0.42-0.71)	0.67 (0.58-0.76)
Oesophagus	MST	0.66 (0.52-0.80)	0.66 (0.51-0.78)	0.67 (0.53-0.82)	0.35 (0.16-0.54)	0.56 (0.44-0.68)
	NUTRISCORE	0.61 (0.47-0.76)	0.41 (0.20-0.61)	0.68 (0.53-0.82)	0.35 (0.16-0.53)	0.55 (0.43-0.67)
Stomach	MST	0.64 (0.56-0.73)	0.49 (0.34-0.64)	0.79 (0.71-0.87)	0.31 (0.18-0.44)	0.60 (0.53-0.68)
	NUTRISCORE	0.59 (0.50-0.67)	0.51 (0.36-0.66)	0.78 (0.70-0.87)	0.29 (0.17-0.41)	0.57 (0.49-0.64)
Small intestine	MST	0.39 (0.16-0.61)	0.63 (0.29-0.96)	0.54 (0.27-0.81)	0.48 (0.20-0.75)	0.50 (0.29-0.71)
	NUTRISCORE	0.22 (0.03-0.41)	0.75 (0.44-1.00)	0.50 (0.15-0.85)	0.46 (0.20-0.73)	0.47 (0.26-0.68)
Digestive lymphomas	MST	0.43 (0.23-0.64)	0.79 (0.61-0.97)	0.67 (0.43-0.91)	0.59 (0.40-0.79)	0.62 (0.47-0.76)
	NUTRISCORE	0.39 (0.19-0.59)	0.79 (0.61-0.97)	0.64 (0.39-0.90)	0.58 (0.39-0.77)	0.60 (0.45-0.74)
Pancreas	MST	0.63 (0.56-0.71)	0.55 (0.43-0.67)	0.77 (0.70-0.84)	0.38 (0.27-0.50)	0.61 (0.55-0.67)
	NUTRISCORE	0.59 (0.52-0.67)	0.58 (0.46-0.70)	0.77 (0.70-0.85)	0.37 (0.26-0.48)	0.59 (0.52-0.65)

MST: Malnutrition screening tool; PPV: Positive predictive value; NPV: Negative predictive value

Table S2. MST and NUTRISCORE diagnostic capabilities based on tumor location: Medium nutritional risk tumors.

Tumor location	Tool	Sensitivity (CI95%)	Specificity (CI95%)	PPV (CI95%)	NPV (CI95%)	Accuracy (CI95%)
Medium nutritional risk						
Lung	MST	0.55 (0.49-0.61)	0.75 (0.70-0.80)	0.71 (0.65-0.77)	0.60 (0.55-0.64)	0.64 (0.60-0.68)
	NUTRISCORE	0.36 (0.30-0.41)	0.87 (0.82-0.91)	0.75 (0.68-0.82)	0.54 (0.50-0.59)	0.60 (0.56-0.64)
Ovary	MST	0.49 (0.39-0.59)	0.77 (0.69-0.85)	0.69 (0.58-0.79)	0.59 (0.51-0.67)	0.63 (0.57-0.70)
	NUTRISCORE	0.19 (0.12-0.27)	0.96 (0.92-1.00)	0.83 (0.68-0.98)	0.53 (0.46-0.61)	0.57 (0.50-0.63)
Endometrium	MST	0.40 (0.23-0.58)	0.73 (0.57-0.90)	0.55 (0.34-0.75)	0.60 (0.44-0.76)	0.58 (0.46-0.70)
	NUTRISCORE	0.27 (0.11-0.43)	0.86 (0.74-0.99)	0.62 (0.35-0.88)	0.59 (0.45-0.74)	0.60 (0.48-0.72)
Biliary tract	MST	0.59 (0.40-0.79)	0.67 (0.46-0.87)	0.64 (0.43-0.85)	0.62 (0.41-0.83)	0.63 (0.49-0.77)
	NUTRISCORE	0.37 (0.19-0.55)	0.81 (0.64-0.99)	0.67 (0.41-0.92)	0.56 (0.38-0.75)	0.59 (0.45-0.74)

Liver	MST	0.44 (0.25-0.64)	0.86 (0.71-1.0)	0.79 (0.57-1.0)	0.58 (0.38-0.77)	0.64 (0.50-0.78)
	NUTRISCORE	0.44 (0.25-0.64)	0.95 (0.87-1.00)	0.92 (0.76-1.00)	0.60 (0.41-0.80)	0.68 (0.55-0.82)
Kidney	MST	0.40 (0.21-0.59)	0.71 (0.49-0.92)	0.67 (0.43-0.91)	0.44 (0.23-0.66)	0.52 (0.37-0.68)
	NUTRISCORE	0.20 (0.04-0.36)	0.94 (0.83-1.00)	0.83 (0.54-1.00)	0.44 (0.24-0.65)	0.50 (0.35-0.65)

MST: Malnutrition screening tool; PPV: Positive predictive value; NPV: Negative predictive value

Table S3. MST and NUTRISCORE diagnostic capabilities based on tumor location: Low nutritional risk tumors.

Tumor location	Tool	Sensitivity (CI95%)	Specificity (CI95%)	PPV (CI95%)	NPV (CI95%)	Accuracy (CI95%)
Low nutritional risk						
Breast	MST	0.41 (0.36-0.46)	0.82 (0.80-0.85)	0.58 (0.53-0.64)	0.70 (0.66-0.73)	0.66 (0.63-0.69)
	NUTRISCORE	0.07 (0.05-0.10)	0.98 (0.97-0.99)	0.72 (0.58-0.86)	0.63 (0.61-0.66)	0.63 (0.61-0.67)
Colorectal	MST	0.54 (0.50-0.59)	0.75 (0.71-0.80)	0.74 (0.69-0.78)	0.57 (0.52-0.61)	0.64 (0.61-0.67)
	NUTRISCORE	0.19 (0.16-0.23)	0.96 (0.93-0.98)	0.85 (0.78-0.92)	0.48 (0.45-0.52)	0.53 (0.50-0.56)
Leukemias	MST	0.51 (0.37-0.66)	0.74 (0.61-0.88)	0.68 (0.52-0.83)	0.59 (0.46-0.73)	0.63 (0.52-0.73)
	NUTRISCORE	0.18 (0.07-0.29)	1.00 (0.92-1.00)	1.00 (0.63-1.00)	0.54 (0.43-0.65)	0.56 (0.48-0.68)
Other lymphoma	MST	0.44 (0.36-0.53)	0.80 (0.73-0.87)	0.67 (0.58-0.77)	0.61 (0.54-0.68)	0.63 (0.57-0.69)
	NUTRISCORE	0.14	0.97	0.83	0.55	0.60
Prostate	MST	0.34 (0.19-0.50)	0.77 (0.62-0.93)	0.60 (0.39-0.81)	0.54 (0.31-0.70)	0.56 (0.42-0.69)
	NUTRISCORE	0.20	1.00	1.00	0.56	0.61
Bladder	MST	0.39 (0.26-0.52)	0.78 (0.66-0.89)	0.68 (0.50-0.85)	0.52 (0.40-0.63)	0.57 (0.47-0.66)
	NUTRISCORE	0.13	0.98	0.88	0.48	0.60
CNS	MST	0.50 (0.01-0.99)	0.80 (0.55-1.00)	0.40 (0.00-0.83)	0.86 (0.67-1.00)	0.74 (0.54-0.94)
	NUTRISCORE	0.25	0.93	0.50	0.82	0.83
Other tumours	MST	0.45 (0.38-0.53)	0.75 (0.69-0.81)	0.60 (0.52-0.69)	0.62 (0.56-0.68)	0.61 (0.57-0.67)
	NUTRISCORE	0.16 (0.10-0.21)	0.97 (0.94-0.99)	0.80 (0.67-0.93)	0.58 (0.53-0.63)	0.60 (0.55-0.65)

MST: Malnutrition screening tool; PPV: Positive predictive value; NPV: Negative predictive value
